# Free fatty acid treatment of mouse preimplantation embryos demonstrates contrasting effects of palmitic acid and oleic acid on autophagy

**DOI:** 10.1152/ajpcell.00414.2021

**Published:** 2022-03-23

**Authors:** Zuleika C. L. Leung, Basim Abu Rafea, Andrew J. Watson, Dean H. Betts

**Affiliations:** ^1^Department of Obstetrics and Gynaecology, The University of Western Ontario, London, Ontario, Canada; ^2^Department of Physiology and Pharmacology, The University of Western Ontario, London, Ontario, Canada; ^3^The Children’s Health Research Institute – Lawson Health Research Institute, London, Ontario, Canada

**Keywords:** autophagy, preimplantation development, preimplantation embryos, nonesterified fatty acids, obesity

## Abstract

Treatment of mouse preimplantation embryos with elevated palmitic acid (PA) reduces blastocyst development, whereas cotreatment with PA and oleic acid (OA) together rescues blastocyst development to control frequencies. To understand the mechanistic effects of PA and OA treatment on early mouse embryos, we investigated the effects of PA and OA, alone and in combination, on autophagy during preimplantation development in vitro. We hypothesized that PA would alter autophagic processes and that OA cotreatment would restore control levels of autophagy. Two-cell stage mouse embryos were placed into culture medium supplemented with 100 μM PA, 250 μM OA, 100 μM PA and 250 μM OA, or potassium simplex optimization media with amino acid (KSOMaa) medium alone (control) for 18–48 h. The results demonstrated that OA cotreatment slowed developmental progression after 30 h of cotreatment but restored control blastocyst frequencies by 48 h. PA treatment elevated light chain 3 (LC3)-II puncta and p62 levels per cell whereas OA cotreatment returned to control levels of autophagy by 48 h. Autophagic mechanisms are altered by nonesterified fatty acid (NEFA) treatments during mouse preimplantation development in vitro, where PA elevates autophagosome formation and reduces autophagosome degradation levels, whereas cotreatment with OA reversed these PA effects. Autophagosome-lysosome colocalization only differed between PA and OA alone treatment groups. These findings advance our understanding of the effects of free fatty acid exposure on preimplantation development, and they uncover principles that may underlie the associations between elevated fatty acid levels and overall declines in reproductive fertility.

## INTRODUCTION

Obesity is often synonymous with an excess accumulation of lipid that leads to inflammation, metabolic disorder, and cellular stress. It impairs vital bodily systems such as the cardiac (1), immune (2), and endocrine systems (3, 4). Obesity also negatively affects reproductive health in both sexes, and in women, these effects include menstrual abnormalities (5), ovarian dysfunction (6), diminished oocyte quality (7), reduced endometrial sensitivity for implantation (8), and overall, significantly reduced conception rates (9). Along with significant contributions from dietary lipid intakes, obesity results from the accumulation of adipose tissues. Adipose tissues release nonesterified fatty acids (NEFAs) into the circulation to be transported to other cells for energy production (10). Profiling of NEFAs in human serum identified palmitic acid (16:0; PA) and oleic acid (18:1; OA) as the most abundant NEFAs in the circulation, estimated at 23% and 18% of total NEFA, respectively (11). Although early literature suggested that adipose tissue mass correlates with NEFA levels in the circulation (12), studies have challenged this consensus (10, 13). For example, McQuaid et al. (13) reported that obese individuals have a systemic NEFA concentration no different from lean individuals. The rate of lipoprotein lipase activity in obese individuals was in fact lowered, which actually reduces the levels of NEFA released from the adipose tissue (13). Regardless of the conflicting conclusions reported, elevated levels of NEFAs were reported in the reproductive system of obese women. Ovarian follicular fluid samples from women of obese body mass index (BMI) displayed a significantly higher level of NEFAs, including PA and OA, compared with women of normal BMI (14). The measurement of fatty acid levels in the human oviductal fluid is limited in the literature. However, other studies have reported the presence of NEFAs, specifically of PA and OA, in bovine oviductal fluid (15) and mouse oviducts and uterus (16). In mouse reproductive tracts, PA was identified to be 10 times higher in the oviduct compared with the uterus (16). Lipid content in the follicular fluid and oviducts can be easily taken up by the oocytes (through cumulus cells) and embryos, respectively, as evident by the presence of lipid droplets in bovine oocytes and blastocysts (17). With the presence of NEFAs in the follicular fluid, embryos are likely exposed to elevated NEFA levels in the reproductive tracts as well.

Exposure of embryos to high lipid environment leads to higher levels of reactive oxygen species (ROS) and lipid peroxides that can result in lipotoxicity (18). In general, lipotoxicity-induced dysregulation of the endoplasmic reticulum (ER) will promote an imbalance in ER protein folding, resulting in the activation of ER stress response mechanisms such as the unfolded protein response (UPR). UPR is activated to maintain ER homeostasis by removing unfolded proteins and preventing further protein unfolding (19). Under prolonged ER stress, cells may opt to activate apoptosis to prevent damage to the neighboring cells or initiate autophagy to reassert cellular homeostasis and avoid escalation of the stress state (20).

Autophagy is a cellular degradation pathway that is highly conserved in eukaryotes (21). It serves many different functions, including organelle clearance, regulation of development, as well as stress adaptation (22). Autophagy is a three-step process that consists of the formation of autophagosomes (23–25), the maturation of autophagosomes to autolysosomes (26), and the degradation of autolysosome contents into monomeric units for other cellular functioning (27). Generally, autophagy acts to maintain homeostasis in a cell but its function and effects vary considerably across cell types and varying environmental conditions. Autophagy has cytoprotective effects in various health conditions, ranging from preventing protein aggregation in the brain to preventing liver degeneration, among many others (28). These findings propose the potential utilization of autophagic regulation as means of therapeutic options. Studies of autophagic pathways in liver cells and pancreatic cells identified that not only does autophagy plays a role in regulating cell homeostasis, but it also counteracts the lipotoxicity-induced stress in cells of diabetic mouse models, which suggests a potential protective mechanism of autophagy against lipotoxicity (29, 30).

We have recently identified that mouse preimplantation embryos display a dose-dependent reduction in blastocyst development when exposed to PA treatment in culture and this effect of PA is reversed when embryos are cotreated with OA (16). PA treatment resulted in the elevation of ER stress markers that were restored to normal levels after PA + OA cotreatment (16). In close association to ER stress and UPR, autophagy may also be elevated when ER stress is activated in PA-exposed preimplantation embryos. Evidence suggests that autophagy may be beneficial in offsetting the negative effects of a high-fat environment (29, 30) and may play a role in early preimplantation embryo development where embryos are highly susceptible to environmental factors. Although autophagy is a fundamental mechanism that must be activated for successful mammalian embryo preimplantation development to occur (31), we propose that autophagy activation may be a key component of the adaptive mechanisms that preimplantation embryos use to offset the deleterious effects of high free fatty acid level exposure in the reproductive tract. We hypothesized that palmitic acid (PA) treatment would elevate autophagy activation and that oleic acid (OA) cotreatment would restore control levels of autophagy during in vitro mouse preimplantation development. Our outcomes demonstrate that autophagy pathway mechanisms are altered by PA and OA treatment during mouse preimplantation development in vitro. We suggest these findings advance our understanding of the effects of free fatty acid exposure on preimplantation development, as well as uncovering principles that may underlie the associations between high fatty acid levels and overall obesity-induced declination in reproductive fertility.

## MATERIALS AND METHODS

### Mouse Preimplantation Embryo Culture

All experiments were conducted using CD-1 mice from Charles River Laboratories (Senneville, QC, Canada). All mice were handled in accordance with the Canadian Council on Animal Care and Western University’s Animal Care and Use Policies (Protocol No. 2018-075). Mice were housed in conventional housing with 12-h light-dark cycle and access to food and water ad libitum. CD-1 female mice of 4–6 wk old were gonadotrophin superovulated to prepare for mating. Female mice received intraperitoneal (ip) injections of pregnant mare serum gonadotrophin (PMSG, Merck Animal Health, Canada) at 5.0 IU and human chorionic gonadotrophin (hCG, Merck Animal Health, Canada) injections at 5.0 IU 48 h between injections. After hCG injections, the female mice were mated overnight and were checked visually for vaginal plugs the next morning. Female mice that were successfully mated were euthanized by CO_2_ asphyxiation 48 h post-hCG injections and embryos at the two-cell stage were collected immediately after. Pools of 20–30 embryos were randomly allocated to treatment groups at a density of one embryo per 1 µL treatment medium for the appropriate culture period. All experimental treatments were prepared with potassium simplex optimization media with amino acids (KSOMaas, Caisson Labs, Smithfield, UT). Bovine serum albumin (BSA) culture medium was prepared with the stock 20% BSA solution to produce a 1.5% BSA in KSOMaa control medium. The BSA solution was used to conjugate PA and OA stock solutions in a 2:1 fatty acid-to-BSA molar ratio. Embryos were cultured in vitro under 5% CO_2_, 5% O_2_, and 90% N_2_ environment at 37°C for up to 48 h. Embryos were treated in vitro with 100 μM PA, 250 μM OA, 100 μM PA and 250 μM OA, or KSOMaa medium alone (control) for 18, 24, 30, 40, and 48 h. Experiments involving chloroquine (CQ) incorporated 75 μM CQ for the final 2 h at the end of the entire culture treatment period. CQ is an autophagic inhibitor that acts to neutralize acidic contents of the lysosomes (32). Experiments were repeated at a minimum of three replicates.

### Developmental Stage Analysis

Embryos were collected for developmental stage analysis after treatment period. Embryos were viewed under a light dissecting microscope and cell stage progression for each embryo was visually classified based on cavitation and blastomere numbers.

### RNA Isolation and RT-qPCR

An Arcturus PicoPure RNA Isolation Kit (KIT0204; Applied Biosystems, Thermo Fisher Scientific, Waltham, MA) was used for RNA extraction of mouse embryos, according to manufacturer’s instructions. Exogenous luciferase mRNA (L4561; Promega Corporation, Madison, WI) was added into RNA samples based on the number of embryos in each sample (0.025 pg/embryo) as a reference gene for mRNA transcript analysis. The use of exogenous RNA as a reference gene is due to the limitations of commonly used housekeeping genes in developing preimplantation embryos (33). Reverse transcription was performed with Sensiscript RT Kit (205213; Qiagen, Germantown, MD), according to manufacturer’s instructions. Quantitative polymerase chain reaction (qPCR) was performed to assess relative mRNA transcript levels using TaqMan Universal PCR Master Mix (4034437; Applied Biosystems, Thermo Fisher Scientific, Waltham, MA), according to manufacturer’s instructions. A nontranscript control of RNase-free water was included for each gene. The BioRad CFX384 Touch Real-Time PCR Detection System (1855484, BioRad, Hercules, CA) was used to run the qPCR reaction with the following conditions: 95°C for 5 min, then 50 cycles of 95°C for 15 s and 60°C for 1 min. Primer probes for luciferase (Luc) *Luc* (5′ ACGTTCGTCACATCTCAT-3′), Beclin-1 (*Bcln1*; Mm01265461_m1, Thermo Fischer Scientific, Waltham, MA), autophagy related protein 3 (*Atg3*; Mm00471287_m1, Thermo Fischer Scientific, Waltham, MA), autophagy related protein 5 (*Atg5*; Mm00504340_m1, Thermo Fischer Scientific, Waltham, MA), and light chain 3 (*Lc3*; Mm00782868_sH, Thermo Fischer Scientific, Waltham, MA) were used for analysis of autophagy-related genes. mRNA transcript levels were measured using the CFX Maestro 1.1 (RRID: SCR_018057; Bio-Rad, Hercules, CA), and relative mRNA abundance was quantified using the delta-delta cycle threshold (2^−ddCt^) method.

### Immunofluorescence Staining and Confocal Microscopy

Mouse embryos were fixed in 2% paraformaldehyde in phosphate-buffered serum (PBS) and then were stored overnight in Pipes Hepes EGTA buffer (PHEM) (34). The embryos were washed once with PBS before blocking with a blocking buffer containing normal serum and Triton X-100 (648466; MilliporeSigma, Burlington, MA) in PBS. Mouse embryos were washed once, then incubated in primary antibody overnight. Three washes were completed before placing embryos into secondary antibody for incubation overnight. One hour of incubation with 4′,6-diamidino-2-phenylindole (DAPI) and phalloidin stain was followed by two washes. Normal donkey serum (Jackson ImmunoResearch, West Grove, PA), anti-LC3A/LC3B polyclonal antibody (1:200; PA1-16931; RRID: AB_2137583; Invitrogen, Thermo Fisher Scientific, Waltham, MA), and Alexa Fluor 488 Donkey anti-Rabbit IgG secondary antibody (1:600; R37118; RRID: AB_2556546; Invitrogen, Thermo Fisher Scientific, Waltham, MA) were used for protein localization of LC3-I and LC3-II. Normal donkey serum, anti-p62/SQSTM1 antibody (1:200; H00008878-M01; RRID: AB_437085; Abnova, Taipei, Taiwan), and Alexa Fluor 488 Donkey anti-Mouse IgG secondary antibody (1:400; R37114; RRID: AB_2556542; Invitrogen, Thermo Fisher Scientific, Waltham, MA) were used for localization and quantification of p62. In the experiments measuring the level of autophagosome maturation and degradation, embryos were incubated with LysoTracker Red DND-99 dye (500 µM; L7528; Invitrogen, Thermo Fisher Scientific, Waltham, MA) for 30 min before fixation. Z-stack images were captured of each embryo at 5 µm intervals using the ×25 objective lens on a ZEISS LSM800 AiryScan confocal microscope (Schulich Imaging Core Facility, Western University, London, ON, Canada). Images for all treatment groups within the same replicate were captured using the same laser and intensity settings. Images captured from confocal microscopy were processed through ZEISS ZEN 3.1 (blue edition; RRID: SCR_013672; Carl Zeiss Microscopy, Jena, Germany) and ImageJ (National Institutes of Health) for image export. Puncta structures (of LC3-II) were defined and counted using the “analyze particle” function of ImageJ (National Institutes of Health) based on pixel area of 9 pixel^2^. Cell numbers were counted manually. Colocalization experiment measured overlapping signal from multichannel images via the RG2B colocalization plug-in (35) on ImageJ. Fluorescence intensity was measured in arbitrary units and analyzed through ilastik (V1.3.3; RRID: SCR_015246; open source).

### Statistical Analyses

Statistical analyses were performed using a one-way ANOVA (two-way ANOVA for NEFA treatments on LC3-II time course experiment) and a Tukey’s honestly significant difference (HSD) post hoc test using Prism 8 (RRID: SCR_002798; GraphPad Software, San Diego, CA). Normalization of all treatment groups to control was conducted to highlight NEFA effects on LC3-II puncta count and p62 fluorescence in fold differences. Experiments were performed with a minimum of three biological replicates. *P* values less than 0.05 were considered statistically significant.

## RESULTS

### Effects of PA and OA on Preimplantation Embryo Development

**Figure 1.**
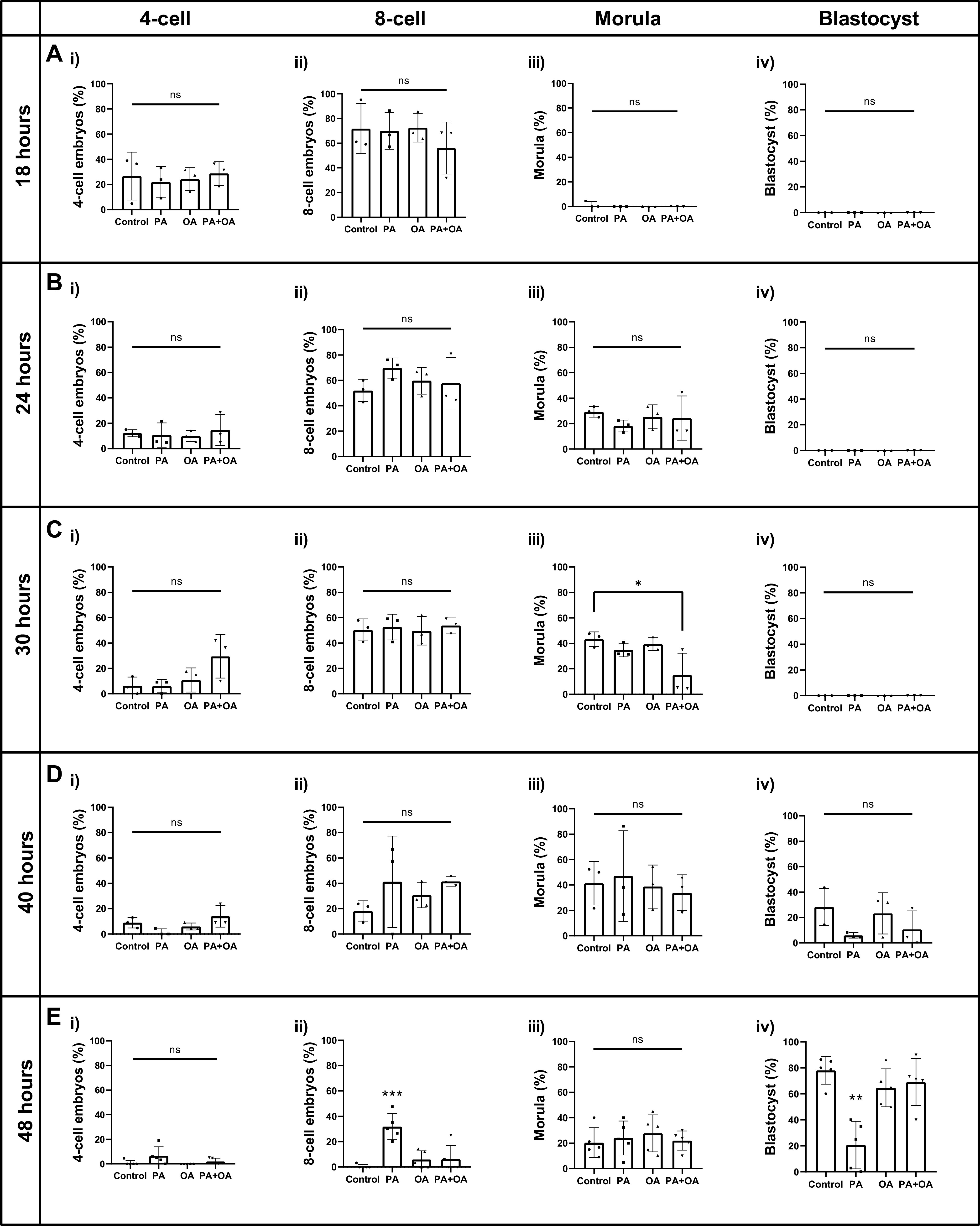
NEFA treatment effects on mouse preimplantation development. Relative percentage (±SD) of 4-cell, 8-cell, morula, and blastocyst stage embryos following treatment of 2-cell mouse embryos in 100 µM PA, 250 µM OA, 100 µM PA and 250 µM OA, or KSOMaa medium alone (control) for 18 (*A*), 24 (*B*), 30 (*C*), 40 (*D*), 48 (*E*) h. *n* > 3, one-way ANOVA with Tukey’s HSD post hoc test. Significant differences are indicated by *P < 0.05, **P < 0.01, ***P < 0.001. By 30 h of treatment (*Ciii*), the percentage of morula stage embryos was significantly lower in the PA + OA group than control (*P* = 0.0317). By 48 h, PA treatment displayed a significant higher percentage of 8-cell embryos [*Eii*; *P* = 0.0001 (vs. control), *P* = 0.0007 (vs. OA), *P* = 0.0008 (vs. PA + OA)] and a significantly lower percentage of blastocysts development [*Eiv*; *P* = 0.0002 (vs. control), *P* = 0.0021 (vs. OA), *P* = 0.0009 (vs. PA+OA)]. The percentage of morula and blastocysts development in the PA + OA group no longer varied significantly from control at 48 h. HSD, honestly significant difference; KSOMaa, potassium simplex optimization media with amino acid; NEFA, nonesterified fatty acid; ns, not significant; OA, oleic acid; PA, palmitic acid.

We recently reported that treatment with 100 µM PA significantly reduces mouse blastocyst development in vitro whereas coculture with 250 µM OA rescues this PA-induced effect resulting in control levels of blastocyst development (16). To uncover the temporal developmental window for the activation of OA reversal of PA treatment, we first dissected the preimplantation developmental time courses for control, PA, and OA treatments alone, and for a combined PA + OA treatment following 18, 24, 30, 40, and 48 h of treatment from the two-cell stage. PA and OA doses were determined following dose response experiments as described previously (16). No significant differences in developmental progression were observed between treatment groups after 18 and 24 h (Fig. 1, *A* and *B*). However, by 30 h of treatment, the percentage of morula stage embryos in the PA + OA coculture group was significantly lower than the control group (Fig. 1*Ciii*; *P* = 0.0317), whereas morula development was similar in PA- and OA-alone groups. This reduction in the morula stage embryos at the 30-h time point suggests that PA + OA combination treatment slows preimplantation developmental progression. However, at the 40-h time point, no significant differences were observed across treatment groups for all stages, including the morula stage (Fig. 1*D*). At the 48-h treatment time, the PA-alone group displayed a significantly higher proportion of eight-cell stage embryos than other treatment groups [Fig. 1*Eii*; *P* = 0.0001 (vs. control), *P* = 0.0007 (vs. OA), *P* = 0.0008 (vs. PA + OA)] along with a significant reduction in blastocyst development [Fig. 1*Eiv*; *P* = 0.0002 (vs. control), *P* = 0.0021 (vs. OA), *P* = 0.0009 (vs. PA + OA)], which was consistent with our previous findings (16). Blastocyst development was similar in the OA-only and PA + OA combination group compared to nontreated controls (Fig. 1*Eiv*). These outcomes indicate that PA treatment exerts an early and rapid impact on embryo development, and that OA rescue of this PA influence requires 30–40 h of cotreatment to alleviate the restrictive influence of PA treatment on preimplantation development.

#### Autophagy pathway transcript profile throughout mouse preimplantation development.

**Figure 2.**
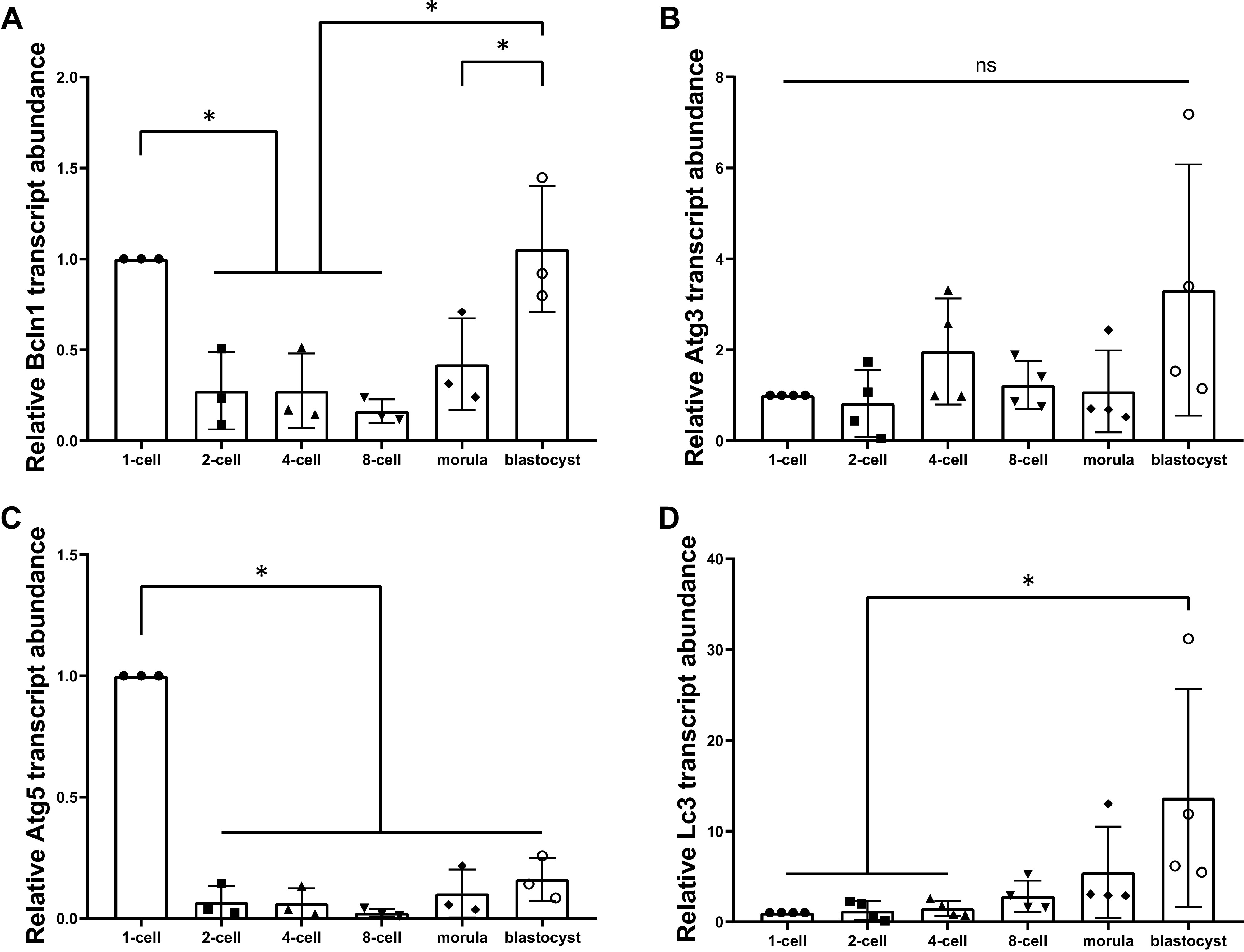
Autophagic marker transcript abundance across mouse preimplantation development. Relative autophagic marker transcript levels (±SD) of preimplantation mouse embryos at 1-cell, 2-cell, 4-cell, 8-cell, morula, and blastocyst stage. *n* > 3, one-way ANOVA with Tukey’s HSD post hoc test. Significant differences are indicated by *P < 0.05. *A*: *Bcln1* relative transcript abundance was significantly decreased at the first cleavage (2-cell) stage and maintained at low levels before returning to the blastocyst stage (*P* < 0.05). *B*: relative *Atg3* transcript levels did not significantly differ across any preimplantation development stages. *C*: relative *Atg5* transcript abundance levels significantly dropped from the zygote stage and stayed at a low relative level throughout preimplantation development (*P* < 0.05). *D*: relative *Lc3* transcript levels significantly increased from the 4-cell stage to the blastocyst stage (*P* < 0.05). *Atg*, autophagy related protein; *Bcln1*, Beclin-1; HSD, honestly significant difference; *Lc3,* light chain 3; ns, not significant.

Next, we characterized the relative mRNA transcript levels of four key autophagy pathway members that participate in autophagosome formation at each stage of mouse preimplantation development. The relative transcript abundances of *Bcln1*, *Atg3*, *Atg5*, and *Lc3* were evaluated after normalization to exogenous control luciferase mRNA levels. *Bcln1* relative transcript abundance was significantly decreased at the first cleavage (2-cell) stage then maintained at relatively low levels until the morula stage, and then returned to one-cell zygote levels by the blastocyst stage [Fig. 2*A*; *P* = 0.0132 (1-cell vs. 2-cell)]. This pattern of mRNA expression follows the typical zygotic genome activation (ZGA) expression pattern as *Bcln1* mRNA of maternal origin degrades during early stages (31, 36, 37). Similarly, relative *Atg5* transcript abundance levels significantly dropped from the zygote stage by more than fivefold after the first cleavage but stayed at a low relative level throughout preimplantation development [Fig. 2*C*; *P* < 0.001 (1-cell vs. 2-cell)]. Relative *Atg3* transcript levels did not significantly differ across any preimplantation development stages (Fig. 2*B*), but *Atg3* mRNA was readily detected in all embryonic stages. In contrast, relative *Lc3* transcript levels significantly increased from the zygote stage to reach a 15-fold increase over the one-cell levels at the blastocyst stage [Fig. 2*D*; *P* = 0.0377 (1-cell vs. blastocyst)].

#### Autophagy pathway transcript profile after PA and OA treatment.

**Figure 3.**
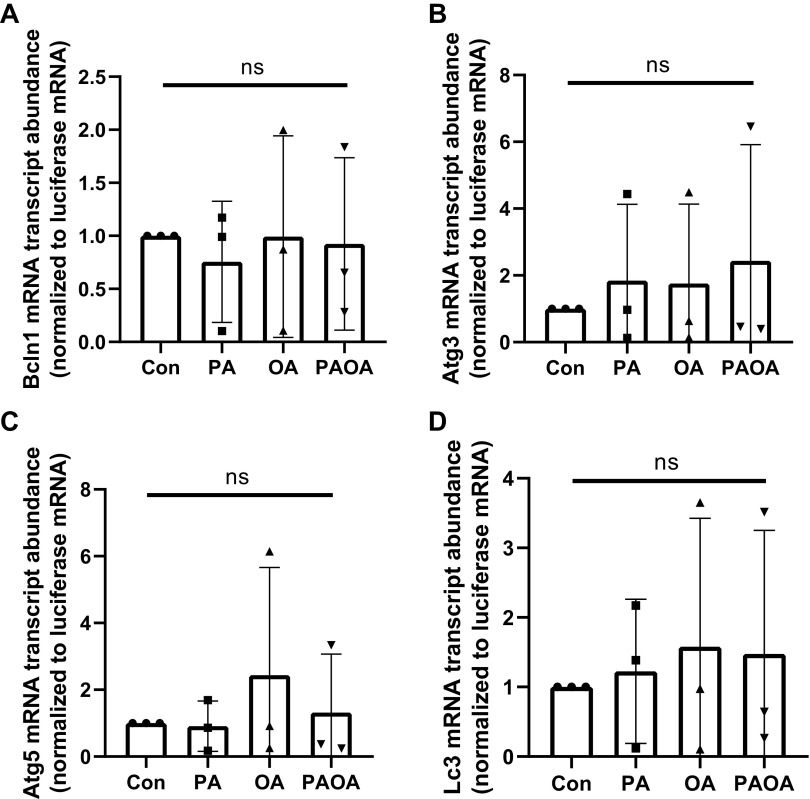
Autophagic marker transcript levels of mouse preimplantation embryos following 48 h of NEFA treatments. Relative transcript levels (±SD) for autophagic marker transcripts in preimplantation mouse embryos treated with 100 µM PA, 250 µM OA, 100 µM PA and 250 µM OA, or KSOMaa medium alone (control) for 48 h. *n* = 3, one-way ANOVA. Relative transcript levels for *Bcln1 (A)*, *Atg3 (B)*, *Atg5 (C)*, and *Lc3 (D)* did not vary significantly across treatment groups compared with controls. *Atg*, autophagy related protein; *Bcln1*, Beclin-1; Con, control; KSOMaa, potassium simplex optimization media with amino acid; *Lc3,* light chain 3; NEFA, nonesterified fatty acid; ns, not significant; OA, oleic acid; PA, palmitic acid.

Interestingly, following PA and OA treatment alone and PA + OA combination treatment, no significant differences in *Bcln1*, *Atg3*, *Atg5*, or *Lc3* mRNA relative transcript abundances were observed after 48 h of treatment (Fig. 3, *A*–*D*). These results suggests that NEFA treatment did not affect the relative transcriptional patterns and half-lives of the selected autophagic markers. This is not unexpected as this outcome has been similarly shown in other cell types (38, 39).

#### LC3-II puncta accumulation during mouse preimplantation development and after PA and OA treatment.

**Figure 4.**
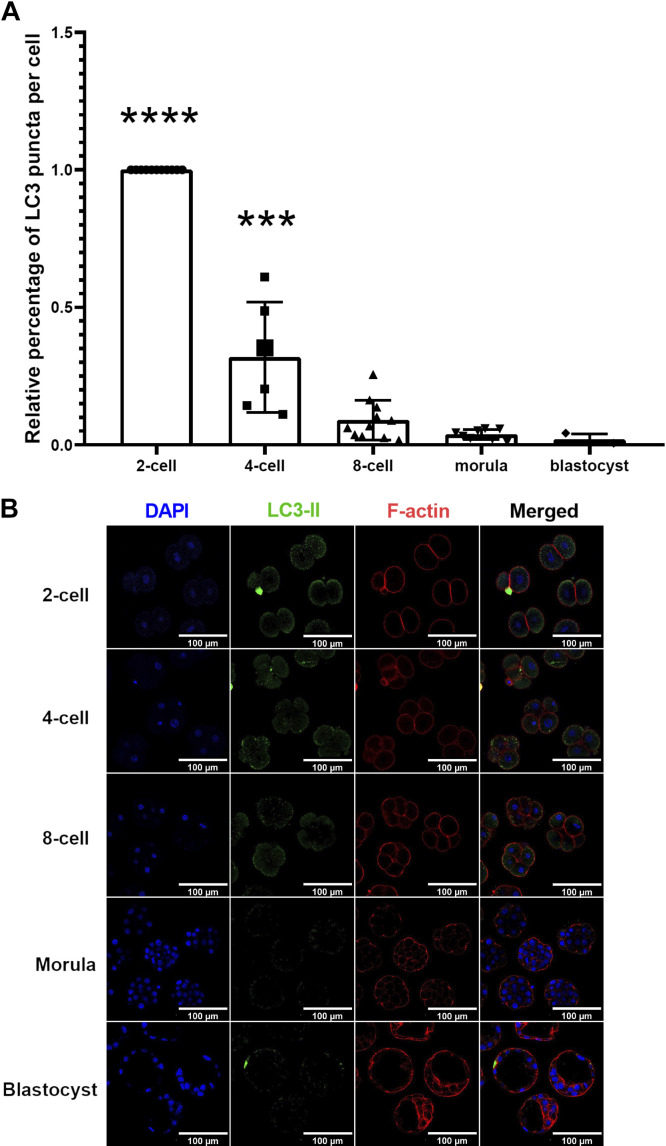
Autophagy marker of LC3-II across mouse preimplantation embryo development. *A*: relative percentage of LC3-II aggregate puncta formation per cell (±SD) at each stage of preimplantation mouse embryos. *n* > 3, one-way ANOVA and Tukey’s HSD post hoc test. Significant differences are indicated by ***P < 0.01 and ****P < 0.0001. Relative percent aggregate puncta formation of LC3-II per cell significantly declined from 2-cell to 4-cell stages and maintained at a low level at all later stages (*P* < 0.01). *B*: representative images of LC3-II puncta at each stage of embryo development. Scale bars = 100 µm. HSD, honestly significant difference; LC3, light chain 3.

Cells exposed to autophagic stimuli initiate autophagy by recruiting activated LC3, also known as LC3-II, to autophagosome membranes, exhibiting a characteristic pattern of LC3-II puncta that represents the presence of autophagosomes (40). We, therefore, investigated the accumulation of LC3-II puncta throughout preimplantation development in control, nontreated embryos first. The analysis of LC3-II puncta per cell by fluorescence microscopy revealed that relative levels of LC3-II protein expression significantly decreased after the two-cell stage, to represent a 100-fold decrease in LC3-II puncta count per cell by the blastocyst stage [Fig. 4, *A* and *B*, *P* < 0.0001 (2-cell vs. blastocyst)].

**Figure 5.**
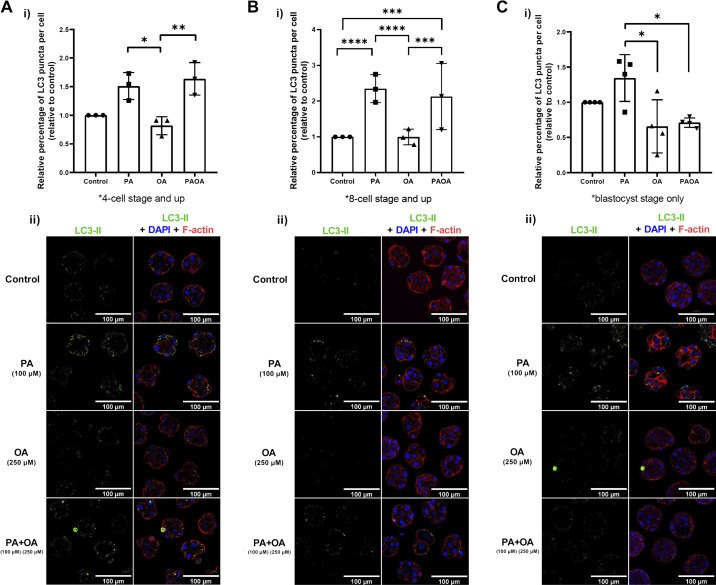
LC3-II puncta per cell in NEFA-treated embryos after 30–48 h of NEFA treatment. Relative percent aggregate of LC3-II per cell (±SD) of 2-cell mouse embryos after exposure to 100 µM PA, 250 µM OA, 100 µM PA and 250 µM OA, or KSOMaa medium alone (control) for 30, 40, and 48 h. Stage-specific embryos from each time point were assessed. *n* > 3, two-way ANOVA with Tukey’s HSD post hoc test. Significant differences are indicated by *P < 0.05, **P < 0.01, ***P < 0.001, and ****P < 0.0001. *A*: at the 30-h time point, PA and PA + OA groups resulted in significantly more LC3-II puncta accumulated per cell than OA-alone group [*P* = 0.0370 (vs. PA); *P* = 0.0098 (vs. PA + OA)]. *B*: at the 40-h time point, both PA and PA + OA groups sustained a significantly higher LC3-II puncta aggregation per cell than control [*P* < 0.0001 (vs. PA); *P* = 0.0002 (vs. PA + OA)] and OA-alone groups [*P* < 0.0001 (vs. PA); *P* = 0.0002 (vs. PA + OA)]. *C*: after 48 h of NEFA exposure, the PA-alone group resulted in a significantly higher LC3-II puncta count per cell compared with both the OA-alone group (*P* = 0.0131) and the PA + OA combination group (*P* = 0.0251). Representative images of LC3-II puncta aggregate after NEFA treatments for 30 (*Aii*), 40 (*Bii*), and 48 h (*Cii*) of exposure. Scale bars = 100 µm. HSD, honestly significant difference; KSOMaa, potassium simplex optimization media with amino acid; LC3, light chain 3; NEFA, nonesterified fatty acid; OA, oleic acid; PA, palmitic acid.

Following the validation of our method, we proceeded to determine the impact of PA and OA treatment alone and in combination on the percentage of LC3-II puncta per cell following 18, 24, 30, 40 and 48 h of treatment. At both 18- and 24-h. treatment times, no significant differences in LC3-II puncta per cell were observed in the PA and OA group, alone and in combination, from that observed in the control group (data not shown). Significant differences in LC3-II puncta counts per cell were first observed after 30 h of NEFA exposure. Two-cell mouse embryos treated with PA for 30 h displayed a significant increase in LC3-II puncta counts per cell compared with treatment with OA for the same time interval (Fig. 5*A*; *P* = 0.0370). Likewise, treatment of two-cell embryos with the PA + OA combination treatment for 30 h also significantly increased LC3-II puncta count per cell compared with OA-alone treatment (Fig. 5*A*; *P* = 0.0098). No significant differences were observed between PA-alone and PA + OA combination group (Fig. 5*A*). Two-cell mouse embryos treated with PA for 40 h also displayed a significant increase in LC3-II puncta per cell by more than twofold compared with nontreated controls (Fig. 5*B*; *P* < 0.0001). The same relative increase in LC3-II puncta per cell was also observed in two-cell mouse embryos treated with the PA + OA cotreatment for 40 h compared with control (Fig. 5*B*; *P* < 0.001). Consistent with other time intervals, treatment of two-cell mouse embryos with PA for 48 h significantly increased LC3-II puncta per cell compared with treatment with OA alone (Fig. 5*C*; *P* = 0.0131). However, surprisingly and in contrast to previous time points, two-cell mouse embryo treated with PA + OA in combination for 48 h resulted in no significant difference in LC3-II puncta count per cell than the OA-alone group (Fig. 5*C*). At 48 h, the PA + OA cotreatment also resulted in a significant reduction in LC3-II puncta count per cell than that observed for the PA-only treatment (Fig. 5*C*; *P* = 0.0251). This result suggests that the addition of OA in the coculture group relieved PA-treated embryos from the elevated levels of autophagy observed, but only after 48 h of treatment.

#### Effects of PA and OA treatment on p62 accumulation.

**Figure 6.**
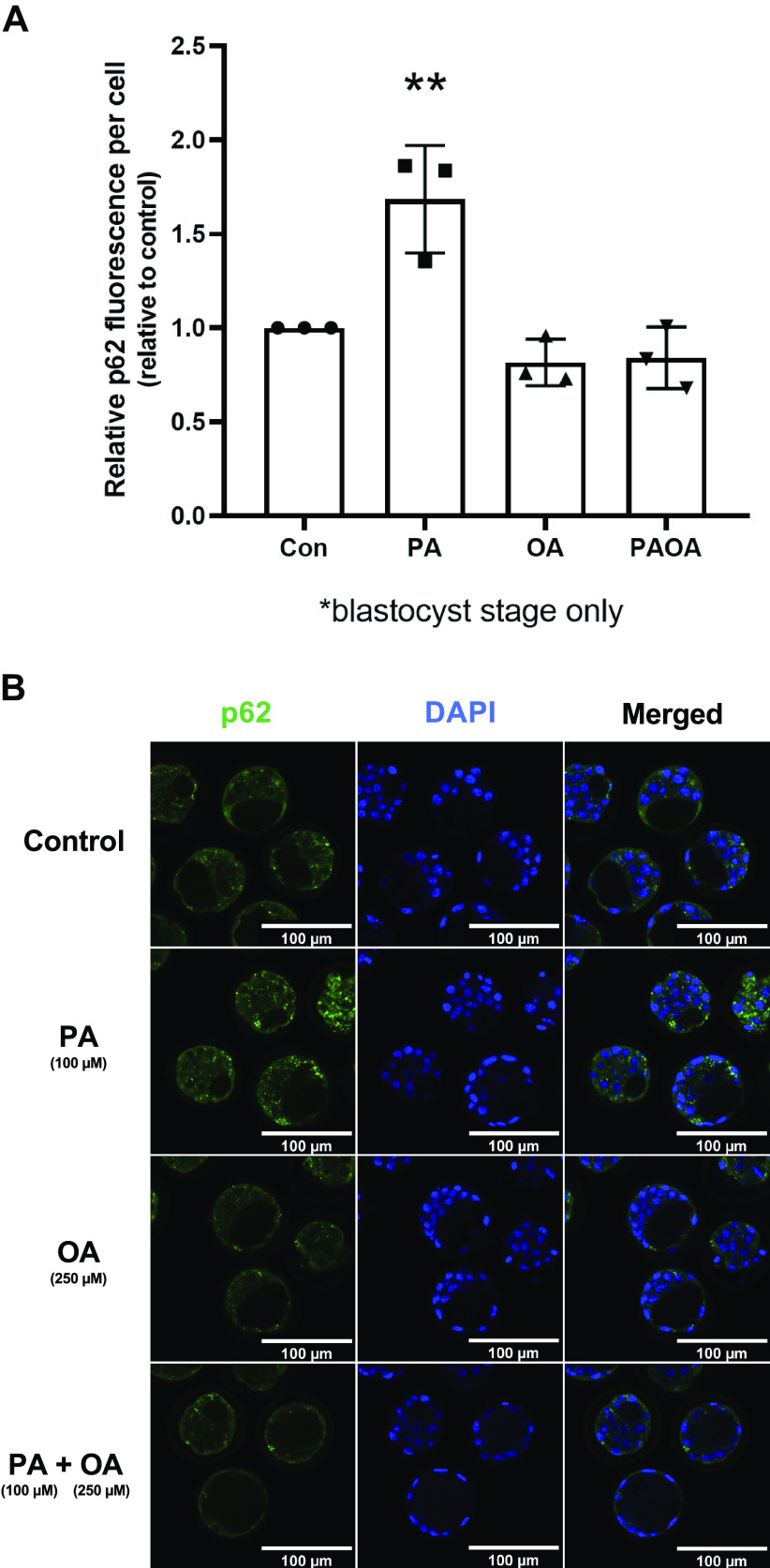
Immunofluorescence intensity of p62 in preimplantation embryos after 48 h of NEFA treatment. Relative p62 immunofluorescence intensity per cell (±SD) of 2-cell mouse embryos after exposure to 100 µM PA, 250 µM OA, 100 µM PA and 250 µM OA, or KSOMaa medium alone (control) for 48 h. Only blastocyst embryos were assessed. *n* = 3, one-way ANOVA with Tukey’s HSD post hoc test. Significant differences are indicated by **P < 0.01. *A*: after 48 h of NEFA exposure, the PA-alone group resulted in a significantly higher LC3-II puncta count per cell compared with the control group (*P* = 0.0062), the OA-alone group (*P* = 0.0014), and the PA + OA combination group (*P* = 0.0017). *B*: representative images of p62 immunofluorescence after 48 h of exposure to NEFA treatments. Scale bars = 100 µm. Con, control; HSD, honestly significant difference; KSOMaa, potassium simplex optimization media with amino acid; LC3, light chain 3; NEFA, nonesterified fatty acid; OA, oleic acid; PA, palmitic acid.

To corroborate the autophagic effects of NEFA treatments on preimplantation embryos, we also evaluated p62 levels [sequestome 1 (p62/SQSTM1)]. p62/SQSTM1 plays an important autophagic role as it brings ubiquitinated proteins into autophagosomes for degradation (41). After 48 h of NEFA treatments, relative p62 immunofluorescence intensity per cell was significantly greater in the PA-treated embryos than embryos from other treatment groups [Fig. 6, *A* and *B*; *P* = 0.0062 (vs. control), *P* = 0.0014 (vs. OA), *P* = 0.0017 (vs. PA + OA)], whereas no significant differences were found between control and PA + OA combination treatment groups. These outcomes confirm that PA treatment exacerbates preimplantation embryo autophagy. Furthermore, the supplementation of OA in PA treatment alleviates preimplantation embryos from PA treatment-induced changes in autophagy levels. Although PA and OA treatments did not affect autophagy pathway constituent relative to mRNA levels after 48 h of treatment time, these treatments do affect LC3-II puncta numbers and p62 levels, indicating differential effects of PA and OA on autophagy regulation during mouse preimplantation development.

#### Effects of PA and OA on autophagosome formation.

**Figure 7.**
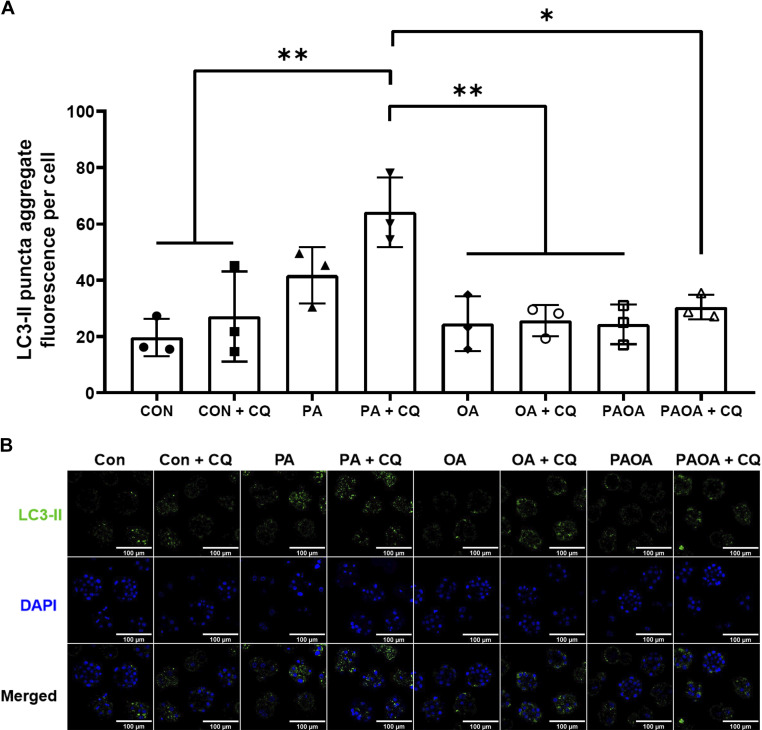
Autophagosome formation of embryos after NEFA ± CQ treatment for 40 h. *A*: LC3-II puncta aggregate fluorescence per cell (±SD) after 40 h of treatment with 100 µM PA, 250 µM OA, 100 µM PA and 250 µM OA, or KSOMaa medium alone (control), with 75 µM chloroquine (CQ) treatment for the last 2 h. Only embryos of 8-cell stage and later were included. *n* = 3, one-way ANOVA and Tukey’s HSD post hoc test. Significant differences are indicated by *P < 0.05 and **P < 0.01. After CQ treatment, fluorescence of LC3-II puncta aggregate per cell of PA group was significantly higher than other treatment groups [*P* = 0.0047 (vs. control + CQ); *P* = 0.0032 (vs. OA + CQ); *P* = 0.0106 (vs. PA + OA + CQ)], suggesting a higher level of autophagosome formation in the PA group. *B*: representative images of LC3-II aggregate puncta formation after NEFA ± CQ treatment for 40 h. Scale bars = 100 µm. Con, control; HSD, honestly significant difference; KSOMaa, potassium simplex optimization media with amino acid; LC3, light chain 3; NEFA, nonesterified fatty acid; OA, oleic acid; PA, palmitic acid.

We next used an autophagic flux analysis to reveal the level of autophagosome formation in PA- and OA- treated mouse embryos with the use of an autophagy inhibitor, CQ. CQ is an autophagic inhibitor that prevents autophagosome-lysosome fusion and degradation (32, 42), thus the incubation of preimplantation embryos with CQ results in the buildup of autophagosomes in the cytosol, allowing the measurement of autophagosome formation. After conducting concentration response and time course experiments, we determined that treatment of mouse embryos with 75 µM CQ for the last 2 h of the treatment period, was sufficient to allow for detection of significant differences in LC3-II puncta cell counts (data not shown). This experimental protocol and design also did not negatively impact embryo morphology between CQ-treated blastocysts and nontreated controls (data not shown). Mouse preimplantation embryos at the two-cell stage were treated with NEFA for 40 h, with 75 µM CQ for the last 2 h of a treatment period to assess autophagic flux. In all CQ treatment groups, no significant difference in the levels of LC3-II puncta fluorescence per cell was observed compared with its corresponding CQ-free treatment group (Fig. 7, *A* and *B*). However, the PA + CQ group displayed a significant increase in fluorescence signal of LC3-II puncta aggregates per cell over that of all other non-PA treatment groups. Specifically, treatment of PA + CQ resulted in approximately a twofold increase in fluorescence signal to that observed for the CON + CQ group (Fig. 7, *A* and *B*; *P* = 0.0047), as well as OA + CQ (Fig. 7, *A* and *B*; *P* = 0.0032) and PA + OA + CQ groups (Fig. 7, *A* and *B*; *P* = 0.0106). Such difference in LC3-II puncta fluorescence between PA + CQ group and non-PA groups is indicative of a significant increase in autophagosome formation in the PA treatment than that of other treatments. No significant differences in fluorescence intensity of LC3-II puncta aggregates per cell were observed between CON + CQ, OA + CQ, and PA + OA + CQ groups (Fig. 7, *A* and *B*). Following the blockade of autophagosome degradation for 2 h with CQ, the addition of OA in PA treatment negated PA-induced elevation of autophagosome formation after 40 h of exposure.

#### Effects of PA and OA on autophagosome maturation and degradation.

**Figure 8.**
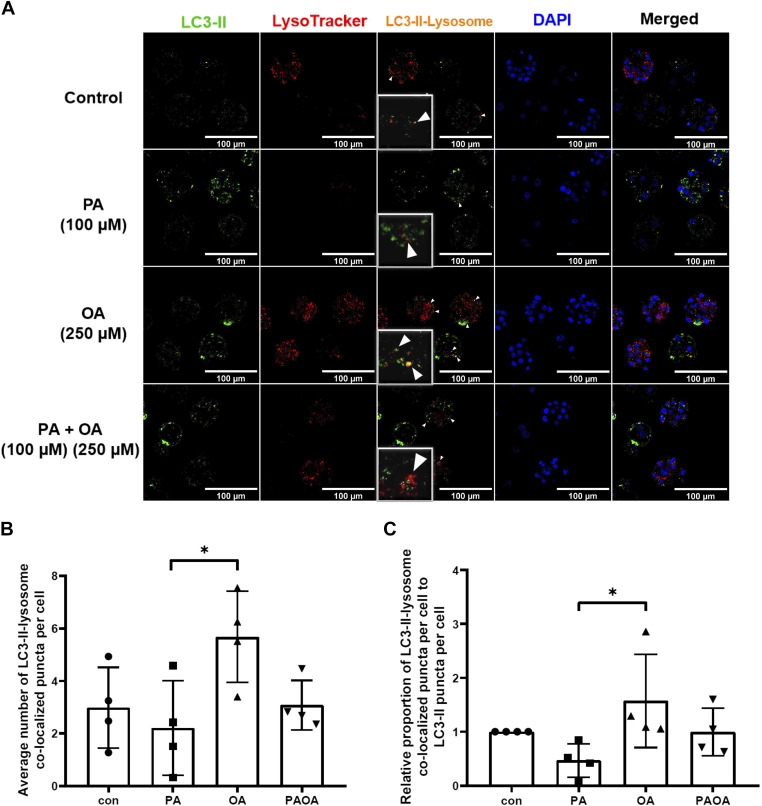
Autophagosome-lysosome colocalization in mouse preimplantation embryos after 40 h of NEFA treatments. LC3-II puncta that are colocalized with lysosomes were quantified after treatment of mouse embryos with 100 µM PA, 250 µM OA, 100 µM PA and 250 µM OA, or KSOMaa medium alone (control) for 40 h. *n* = 4, one-way ANOVA and Tukey’s HSD post hoc test. Significant differences are indicated by *P < 0.05. Only embryos of 8-cell stage and later were included. *A*: representative images of LC3-II-lysosome colocalization in mouse embryos after NEFA exposure. Arrowheads indicate for LC3-II-lysosome colocalized puncta. Scale bars = 100 µm. *B*: average number of LC3-II-lysosome colocalized puncta per cell (±SD) after NEFA treatments for 40 h. PA-treated group resulted in a significantly lowered number of LC3-II-lysosome colocalized puncta per cell than OA-alone group (*P* = 0.0346). *C*: relative proportion of LC3-II-lysosome colocalized puncta per cell to total LC3-II puncta per cell (±SD) of preimplantation mouse embryos after exposure to 40 h of NEFA treatments. Exposure to 40 h of PA treatment results in a significantly lower proportion of autolysosome compared with OA-only group (*P* = 0.0414). Con, control; HSD, honestly significant difference; KSOMaa, potassium simplex optimization media with amino acid; LC3, light chain 3; NEFA, nonesterified fatty acid; OA, oleic acid; PA, palmitic acid.

**Figure 9.**
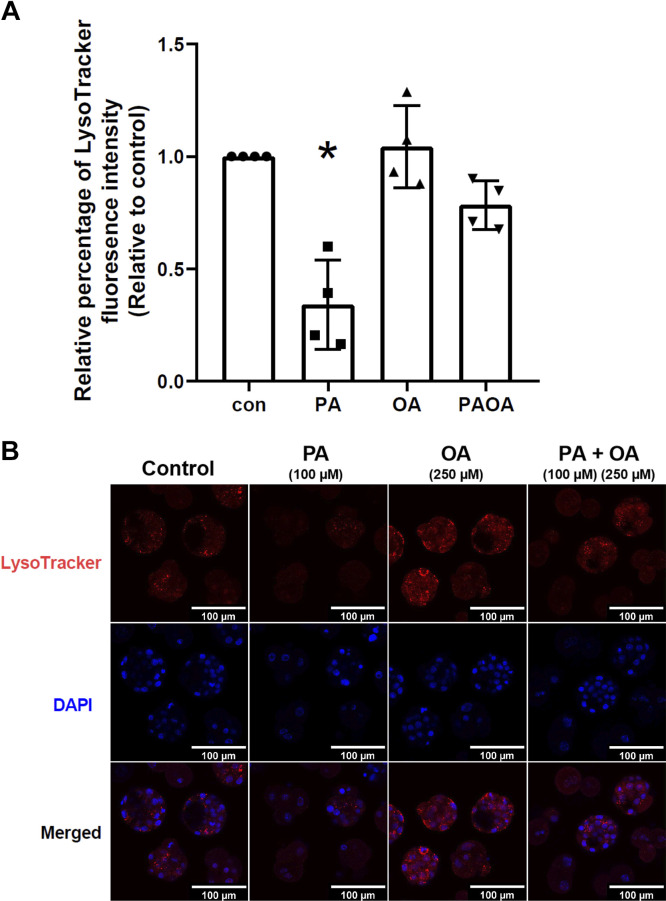
Relative level of LysoTracker fluorescence in mouse preimplantation embryos after NEFA treatments for 40 h. Fluorescence of LysoTracker were quantified in mouse preimplantation embryos after treatment with 100 µM PA, 250 µM OA, 100 µM PA and 250 µM OA, or KSOMaa medium alone (control) for 40 h. *n* = 4, one-way ANOVA and Tukey’s HSD post hoc test. Significant differences are indicated by *P < 0.05. Only embryos of 8-cell stage and later were included. *A*: relative level of LysoTracker fluorescence signal (±SD) after 40 h of NEFA treatments. Exposure to 40 h of PA treatment results in a significantly lower LysoTracker signal compared with all other treatment groups, suggesting a lowered lysosomal activity in mouse preimplantation embryos after PA exposure [*P* = 0.0002 (vs. control), *P* < 0.0001 (vs. OA), *P* = 0.0048 (vs. PA + OA)]. *B*: representative images of LysoTracker fluorescence staining in mouse embryos following 40 h of NEFA treatment. Scale bars = 100 µm. con, control; HSD, honestly significant difference; KSOMaa, potassium simplex optimization media with amino acid; NEFA, nonesterified fatty acid; OA, oleic acid; PA, palmitic acid.

The level of autophagosome maturation was estimated by the presence of colocalization of LC3-II puncta with lysosomes (43, 44). Assessment of the average number of LC3-II-lysosome colocalized puncta per cell revealed that mouse embryos exposed to OA for 40 h resulted in an approximately threefold significant increase of colocalized puncta than embryos treated with PA alone (Fig. 8, *A* and *B*; *P* = 0.0346), which suggests that treatment with OA results in more autophagosome maturation than treatment with PA. Treatment with OA also resulted in a significantly higher proportion of autolysosomes per cell than that observed in embryos treated with PA alone (Fig. 8*C*; *P* = 0.0414). Altogether, this outcome strongly suggests that a higher number and proportion of autolysosomes arise in the OA treatment group compared with the PA group. Finally, we investigated the effects of PA and OA treatment on lysosomal activity to assess the level of autophagosome degradation. Comparison of the fluorescence signal of LysoTracker Red across each treatment group revealed that after treatment with PA for 40 h, fluorescence intensity level of LysoTracker was significantly lower compared with all other treatment groups [Fig. 9, *A* and *B*; *P* = 0.0002 (vs. control), *P* < 0.0001 (vs. OA), *P* = 0.0048 (vs. PA + OA)]. This revealed a significantly lower lysosomal activity, and thus lesser autophagosome degradation, in PA-treated embryos. LysoTracker Red fluorescence intensity after 40 h of treatment with OA alone or PA + OA cotreatment resulted in no significant differences from that observed in the control group (Fig. 9, *A* and *B*), suggesting that the addition of OA could restore lysosomal activity and thus reestablish autophagosome degradation in PA-treated preimplantation embryos.

## DISCUSSION

Our study presents outcomes from the NEFA treatment time course on the development of early mouse embryos. PA + OA combination treatment significantly decelerates preimplantation development of two-cell stage mouse embryos after 30 h of treatment but interestingly induced a “catch-up growth” effect that resulted in blastocyst frequencies similar to that of untreated controls. We propose that the decelerated progression and catch-up growth effect may find a basis in differential uptake rates reported for PA and OA at varying embryo cell stages (45). Therefore enhanced OA uptake at the later preimplantation stages (45) may promote this “catch-up growth” via the conversion of OA to oxidative metabolism energy intermediates and phospholipids (45) to provide sufficient structural and energy demands for accelerated growth to the blastocyst stage. Our results support that this process is well underway by 30 h of combined treatment and is certainly well in place by 40 h of cotreatment as developmental stage differences between the untreated control and cotreatment were no longer observed at the 40-h time point and beyond. Altogether, consistent with our previous findings (16), OA exerts an ability to offset PA treatment-induced developmental impairments. It is important to note that embryos display asynchronous cleavage division in treatment pools as they progress toward the blastocyst stage, thus intraassay developmental variation is normal. However, our study has uniquely defined a developmental window between 30 and 40 h of treatment, where OA fully engages in providing this beneficial outcome to the early embryo. Our outcomes have direct application to improving in vitro embryo culture environments, as OA may become an important addition to human embryo culture media that could improve blastocyst development in vitro.

We presented the temporal pattern of LC3-II protein expression after exposure to NEFA treatments throughout mouse preimplantation development. Two-cell mouse embryos exposed to PA-only and PA + OA cotreatment displayed a significant increase in autophagic activation after 30 h of exposure. This outcome likely defines a developmental window when the embryo activates autophagy to reestablish homeostasis. The induction of autophagy, as reflected by LC3-II puncta counts, occurred between 24 and 30 h of PA exposure. To our surprise, exposure to PA + OA cotreatment for 48 h did not maintain this elevation in the LC3-II puncta count that was observed at the 30 and 40 h time points. The evaluation of p62 immunofluorescence signal levels also showed consistent results in which PA + OA cotreatment restored control levels of autophagy after 48 h of cotreatment. We propose that the exponential increase in OA uptake during the morula-blastocyst phase of preimplantation development (45) may contribute to altered energy metabolism that negates PA individual effects and restores appropriate embryonic homeostasis at the later stages of preimplantation development. Significantly larger and increased numbers of lipid droplets have been observed in bovine oocytes (46) and in mouse preimplantation embryos (16) when cotreated with PA and OA in comparison with PA treatment. It is likely that an OA-induced redirection of PA into lipid droplets (as triglycerides) reduces PA metabolism to diacylglycerol (DAG) and ceramide that induces ER stress, and thus, lessens PA-induced effects on autophagosome accumulation to maintain embryo homeostasis. Beginning at the 30-h time point, autophagosomes appear to accumulate in the PA-treated embryos, as reflected by the elevated levels of LC3-II puncta and p62 immunofluorescence at the 48-h time point. The underlying mechanism of PA effects on autophagy in preimplantation embryos is unclear but studies of other cell types could provide mechanistic insights. Exposure of HepG2 cells to PA revealed that autophagy is induced by the protein kinase C (PKC) pathway as the high level of DAG presence from PA exposure elevated PKC-α activation (47). Another study of mouse hepatocytes suggested that autophagy was dependent on the mammalian target of rapamycin (mTOR) pathway, where PA exposure elevated ER stress and reduced lysosomal-associated membrane protein 2 (LAMP2) expression, thus impairing the autophagic mechanisms (48). Further investigation is required to determine if either PKC or mTOR pathway is implicated in mediating PA treatment effects on mouse preimplantation embryos. Regardless, prolonged accumulation of autophagosomes likely results in a cytotoxic response in the embryos that could underlie the early developmental arrest observed in PA-treated embryos. The buildup of autophagosomes in various cell lines with little lysosomal activity was previously reported to result in a significant reduction in cell viability (49). Autophagosome accumulation is evidently closely associated with cytotoxicity.

Autophagic flux is defined as the turnover of autophagosomes with consideration of autophagosome formation and degradation (40). Our study successfully identified NEFA effects on autophagosome formation which was greater after PA treatment compared with controls. Similar reports of PA treatment increasing autophagosome accumulation, by increasing autophagosome formation, were previously described in pancreatic β-cells (50, 51). Furthermore, we observed a higher number and proportion of autolysosomes in mouse preimplantation embryos treated with OA compared with the PA-treated embryos. No differences in autolysosome levels were observed between control and PA-treated embryos, but PA was previously reported to lower Rab7 activity in hypothalamic neuronal cells (52), which interferes with autophagosome transport toward lysosomes. A similar mechanism following PA treatment may be at play in preimplantation embryos as well, but further investigation must be conducted to examine whether Rab7 activity is altered. Although our study did not show specific PA effects on autophagosome maturation in the preimplantation embryos, the measurement of lysosomal activity revealed significant impacts in autophagosome degradation. We observed that lysotracker staining and thus lysosomal activity in PA-treated embryos was significantly lowered, indicating that autophagosome degradation was disrupted. This interruption in lysosomal activity is not limited to preimplantation embryos, as PA treatment also significantly lowered the hydrolase activity of lysosomes in INS1 β-cells (50) as well as lowered LAMP2 levels in mouse hepatocytes (48). On the other hand, the addition of OA in PA treatment restored normal levels of autophagosome formation. It also negated PA effects on lysosomal activity, resulting in a control comparable level of autophagosome degradation in cotreated embryos. The high level of lysosomal activity observed in the OA group suggests that lysosomes may contribute to the dissolution of accumulated lipid droplets via autophagy, a mechanism specifically described as lipophagy (53). Lipophagy is reported in other cell types like hepatocytes, and it contributes to producing energy to counteract PA effects (53, 54). Further investigations into the occurrence of lipophagy during preimplantation development are warranted and required to generate a full understanding of OA treatment mechanisms used by the embryo to alleviate PA treatment effects.

The pattern of *Bcln1* and *Atg5* relative mRNA levels resembled that of many other transcripts expressed during mouse preimplantation development (31, 55–57). This pattern reflects the well-known degradation of maternal transcripts followed by ZGA and a stage-specific increase in relative mRNA transcript levels up to the blastocyst stage (36, 37, 58). Our discovery of the restoration of zygotic *Bcln1* mRNA transcript levels at the blastocyst stage suggests that the embryonic genome is capable of transcribing *Bcln1* during mouse preimplantation development. The inclusion of later-staged blastocysts (E4.5) in our blastocyst groups likely contributed to the elevation of autophagy that was observed, as autophagy evidently increases in both trophoblast and inner cell mass during embryo implantation (59). The steady production of *Atg3* at all preimplantation stages suggests that it is a functional autophagic pathway requirement for development and consistent LC3 activation throughout preimplantation development. We reported a variable *Lc3* mRNA expression pattern from that of previous studies (36, 37), although study design variation exists between studies with regard to the timing and stage of in vitro culture between reports. As in vitro versus in vivo embryo stage differences are observed for phosphatidylinositol 3-kinase (PI3K)-Akt-mTOR pathway constituents’ expression (60), LC3 production and autophagy are expectedly affected. Further studies are required to determine if this notion has merit.

The use of confocal fluorescence microscopy to assess LC3-II puncta that represent autophagosomes is an accessible and powerful method for measuring autophagy (40). Interestingly, the significant decrease in LC3-II puncta counts per cell throughout preimplantation development was opposite to the increase in LC3 mRNA transcript levels observed. Even though LC3-II puncta count per cell decreased as development advanced, embryonic cell number dramatically increased during preimplantation development, which together results in an overall increase in total LC3-II puncta count per embryo as development to the blastocyst stage proceeded. Due to the asymmetric nature of early embryo cleavage divisions, we measured autophagic activity by determining LC3-II puncta per cell as our primary outcome metric. However, since the total LC3-II puncta count per embryo increased as a result of increasing total cell number as development proceed, our observation of increased *Lc3* mRNA by the blastocyst stage is consistent with our overall LC3-II puncta counts.

**Figure 10.**
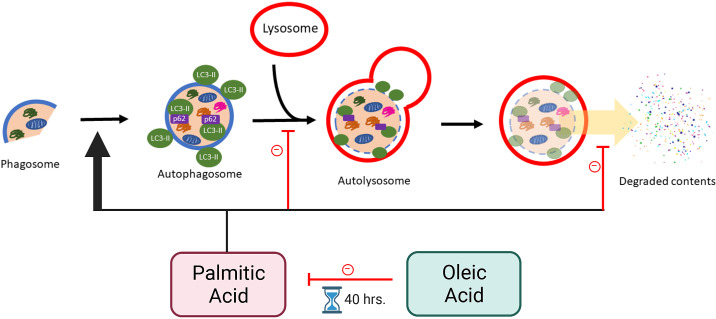
Model of PA and OA treatment effects on autophagy during mouse preimplantation development. Autophagy begins when a phagosome is initiated to form an autophagosome. Phagosomes recruit autophagy-related proteins to direct contents into the phagosome as it elongates and seals off to become an autophagosome. After autophagosome formation is completed, autophagosomes are transported to a lysosome for autophagosome maturation. Fusion of autophagosomes and lysosomes creates an autolysosome. Acidic contents of the lysosome degrade the autophagosome, then releasing the degraded contents into the cytosol for recycling. PA treatment of mouse preimplantation embryos increased autophagosome accumulation by elevating autophagosome formation, reducing autophagosome maturation, and reducing autophagosome degradation by disruption of lysosomal activity. In contrast, OA treatment alone had no obvious effect on these events. However, PA + OA cotreatment restricted PA-induced effects on preimplantation embryo autophagy, but only after 40 h of cotreatment time, resulting in the successful reversal of PA-induced effects by 48 h of exposure. Created with BioRender.com. LC3, light chain 3; OA, oleic acid; PA, palmitic acid.

In summary, we have presented new outcomes from experiments that investigated the effects of PA and OA treatment on preimplantation development and autophagy, specifically on autophagosome formation, maturation, and autophagosome degradation in mouse preimplantation embryos. PA treatment beginning at the two-cell stage impaired blastocyst development by arresting mouse embryos at the four- to eight-cell stage. The significant accumulation of autophagosomes in the later stages of PA-treated embryos was linked to elevated levels of autophagosome formation as well as lowered levels of autophagosome degradation, thus contributing to developmental incompetency in PA-treated embryos. OA treatment beginning at the two-cell stage had no significant effect on preimplantation development, autophagosome formation, or autophagosome degradation. A delay in development was identified at the early cleavage stages of PA + OA cotreated embryos but the affected embryos restored blastocyst development frequency to control levels. A correlation was observed between development and autophagy as the restoration of blastocyst formation coincided with the restoration of autophagy at the later stages of preimplantation embryo development. Interestingly, although high levels of autophagosome accumulation were observed after 40 h of PA + OA cotreatment, levels of autophagosome formation, maturation, and degradation were not significantly different from control at this time point. The 40-h time point seems to fall within the key period of the “catch-up growth,” warranting further experimentation to fully understand NEFA effects on autophagy during preimplantation development. Overall, we demonstrated a key link between embryo development and autophagy, and that autophagic mechanism is altered by NEFA treatments (Fig. 10). These findings advance our understanding of the effects of NEFAs on preimplantation embryo autophagy. They will also guide future investigations that may eventually use autophagic modulation, perhaps by OA treatment, as an option to enhance the developmental success of in vitro produced human preimplantation embryos. It is our goal to assist patients who are seeking help from assisted reproductive technologies for their fertility needs, especially those who are obese.

## GRANTS

This work was supported by a project grant from the Canadian Institutes of Health Research (CIHR) Canada to D.H.B., B.A.R., and A.J.W. Z.C.L.L. was supported by the Children’s Health Research Institute (CHRI) Trainee Award, funded by the Children’s Health Foundation.

## DISCLOSURES

No conflicts of interest, financial or otherwise, are declared by the authors. 

## AUTHOR CONTRIBUTIONS

Z.C.L.L., A.J.W., and D.H.B. conceived and designed research; Z.C.L.L. performed experiments; Z.C.L.L. analyzed data; Z.C.L.L., A.J.W., and D.H.B. interpreted results of experiments; Z.C.L.L. prepared figures; Z.C.L.L. drafted manuscript; Z.C.L.L., A.J.W., and D.H.B. edited and revised manuscript; Z.C.L.L., B.A.R., A.J.W., and D.H.B. approved final version of manuscript. 
